# Assessing negative cognitive style: Development and validation of a Short-Form version of the Cognitive Style Questionnaire

**DOI:** 10.1016/j.paid.2011.11.026

**Published:** 2012-04

**Authors:** Elizabeth Meins, Simon McCarthy-Jones, Charles Fernyhough, Glyn Lewis, Richard P. Bentall, Lauren B. Alloy

**Affiliations:** a Department of Psychology, Durham University, South Road, Durham DH1 3LE, UK; b Academic Unit of Psychiatry, University of Bristol, Bristol BS8 2BN, UK; c Institute of Psychology, Health and Society, University of Liverpool, Liverpool L69 3GL, UK; d Department of Psychology, Weiss Hall 1701 North 13th Street, Philadelphia, PA 19122-6085, United States

**Keywords:** Cognitive style, Depression, Anxiety

## Abstract

The Cognitive Style Questionnaire (CSQ) is a frequently employed measure of negative cognitive style, associated with vulnerability to anxiety and depression. However, the CSQ’s length can limit its utility in research. We describe the development of a Short-Form version of the CSQ. After evaluation and modification of two pilot versions, the 8-item CSQ Short Form (CSQ-SF) was administered to a convenience sample of adults (*N* = 278). The CSQ-SF was found to have satisfactory internal reliability and test–retest reliability. It also exhibited construct validity by demonstrating predicted correlations with measures of depression and anxiety. Results suggest that the CSQ-SF is suitable for administration via the Internet.

## Highlights

► We developed a Short-Form version of the Cognitive Style Questionnaire (CSQ). ► The CSQ Short Form (CSQ-SF) demonstrated satisfactory internal reliability. ► Test–retest reliability was also satisfactory. ► Scores demonstrated predicted correlations with measures of depression and anxiety. ► The CSQ-SF may be a useful research tool in assessing vulnerability to depression.

## 1 Introduction

Identifying why certain individuals may be more vulnerable to depression is an increasingly important research question. The hopelessness theory of depression (Abramson, Metalsky, & Alloy, 1989) proposes that possession of a negative cognitive style increases the probability of depression developing after a negative life event. The cognitive vulnerability engendered by a negative cognitive style arises from an individual tending to make particular kinds of inferences about the causes, consequences, and self-worth implications of negative life events. In particular, a negative cognitive style is defined as the tendency to attribute negative life events to *stable* causes that will persist over time, *global* causes that affect many areas of the individual’s life, and *internal* causes that are inherent to the person (Abramson, Seligman, & Teasdale, 1978), and to infer negative characteristics about oneself and negative consequences about one’s future as a result of the life event. Cross-sectional and prospective studies show relations between negative cognitive style and depression (e.g., Alloy et al., 2000, 2006; Safford, Alloy, Abramson, & Crossfield, 2007). A reliable and valid measurement of cognitive vulnerability is thus of crucial importance to empirical studies in this field (Haeffel et al., 2008).

Negative cognitive style is most commonly assessed using the Cognitive Style Questionnaire (CSQ; Alloy et al., 2000), which was developed from the Attributional Style Questionnaire (Peterson et al., 1982). The CSQ focuses on 24 hypothetical events (12 positive, 12 negative) relating to successes and failures in academic achievement, employment, and interpersonal relationships. For each event, participants are told vividly to imagine themselves in that situation, and then to write down the one major cause of the event. Next, participants are asked to rate the extent to which the named cause was the result of (a) internal versus external factors (i.e., caused by themselves or other people/circumstances), (b) specific versus global factors (whether the cause of the event has implication for all areas of life or only that specific situation), and (c) stable versus unstable factors (whether the cause will persist and always lead to the same outcome in the future). In the final section of the CSQ, participants are asked about the meaning of the event (rather than its cause), rating whether the event (d) means that other negative/positive events will happen to them, (e) means that they are flawed/special in some way, and (f) matters to them.

Despite its observed satisfactory psychometric properties (Alloy et al., 2000; Haeffel et al., 2008), the length of the CSQ is problematic (Haeffel et al., 2008), with participants often taking more than 30 min to complete responses to the 24 hypothetical events. This reduces the potential clinical utility of the measure, and led Haeffel et al. (2008) to conclude that “future research is needed to determine whether a brief version of the CSQ can be created that maintains the reliability and validity of the full scale” (p. 833). The main aim of the present study was to create a Short-Form version of the CSQ with satisfactory psychometric properties.

A second aim was to establish whether the Short-Form CSQ could be reliably administered remotely via the Internet. Jones, Fernyhough, de-Wit, and Meins (2008) reported that administration format (electronic versus paper-and-pen) was not related to participants’ responses on psychopathology questionnaires. We accordingly investigated whether responses on the Short-Form CSQ were related to administration format.

## 2 Method

### 2.1 Participants

The development of our new Short-Form CSQ was an iterative process involving three increasingly refined versions of the CSQ. These versions are termed CSQ-13, CSQ-11, and CSQ-SF, and were administered to three separate sets of participants. A first convenience sample of 249 (160 women) adults with a mean age of 21.7 years (*SD* = 7.05, range = 17–58) completed the CSQ-13. A separate convenience sample of 390 (257 women) undergraduate students (mean age = 20.2 years, *SD* = 1.65, range = 17–32) then completed the CSQ-11. Finally, a new convenience sample of 278 adults (145 women) (mean age = 21.4 years, *SD* = 6.86, range = 18–62) completed the CSQ-SF. Of this sample, 193 participants (102 women), with a mean age of 20.4 years (*SD* = 4.25, range 18–55), went on to complete the Hospital Anxiety and Depression Scale (HADS: Zigmond & Snaith, 1983). Four weeks after the first testing session, 60 of the original participants (54 female), with a mean age of 19.6 years (*SD* = .85, range = 19–24), completed the CSQ-SF for a second time to investigate test–retest reliability. No incentive was offered for participation.

### 2.2 Measures and procedure

The CSQ-13 and CSQ-SF were completed in paper-and-pen format; the CSQ-11 was completed in electronic format. To recruit the latter sample, a circular email was sent out to undergraduates from a wide range of degree programs at a British university, directing them to a website link. The only personal details requested for both formats were age and gender; participants were not screened for psychiatric disorders. The instructions and format of each scenario in the electronic version of the CSQ were identical to those of the corresponding paper-and-pen version.

In the administration of the third and final version (CSQ-SF), a sub-group of participants additionally completed the HADS (Zigmond & Snaith, 1983). This is a brief and psychometrically sound (Zigmond & Snaith, 1983) instrument to measure psychological distress. The HADS contains 14 items and consists of two subscales: anxiety and depression.

### 2.3 Questionnaire design

Our shortening of the CSQ first focused on the requirement to write down a potential cause for each scenario. The named causes are not analyzed and play no role in determining participants’ scores on the CSQ. However, the named cause for each event is repeatedly mentioned in the questions that follow, making the wording of the item lengthy and complex (see Table 1 for an example). In our adaptation of the CSQ, participants were not required to write down a specific cause, but were simply directed to “Think carefully about the reason for [scenario] then answer the questions below”.

Our second adaptation served further to simplify the wording of the individual questions. This was achieved by dividing the internal, stable, global, and self-worth response scales on the original CSQ each into two separate items. For example, the original item shown in Table 1 became the following separate items: (a) my job evaluations in the future will be affected by the same reason that caused this negative evaluation, and (b) the reason for this negative evaluation will not impact on my future job evaluations. The negative consequences item (the likelihood that other negative things would result) was maintained as a single item in the adapted version of the CSQ. As shown in Supplementary Material: Appendix 1, for each scenario, participants rated cognitive style in terms of internality (items 1 and 6), globality (items 2 and 7), stability (items 3 and 8), negative consequences (item 4), and self-worth implications (items 5 and 9). All items were rated using the same 5-point Likert scale ranging from ‘strongly agree’ to ‘strongly disagree’. Items were scored so that higher scores indicated more negative cognitive style.

The third modification involved removing the positive scenarios, thus halving the length of the instrument. Our rationale was that depression is more strongly related to inferences for negative scenarios than those for positive scenarios (Alloy et al., 2000, 2006). Indeed, an *ad hoc* strategy of presenting only the negative scenarios has already been employed in some studies (e.g., Gibb, Alloy, Abramson, Beevers, & Miller, 2004). However, omitting the positive items from the CSQ in the absence of any further adaptations is potentially problematic. Haeffel et al. (2008) identified two reasons for the original inclusion of positive items in the CSQ: (a) to assess the individual’s “enhancing inferential style… the tendency to make stable, global attributions and infer positive consequences and self-worth characteristics for positive (rather than negative) life events” (p. 826), and (b) to reduce the chances of a response set bias. While omission of positive items is unlikely to be problematic if negative cognitive style is the focus of research, response bias remains a potential threat to reliability and validity. Allowing all items to be rated on the same Likert scale enabled us to reduce the probability of response set bias by including reverse-worded items (Cronbach, 1970). Thus, to indicate negative cognitive style consistently, participants would have to agree with some items but disagree with others. Supplementary Material: Appendix 1 shows how reverse-worded items were included to rate a scenario.

The final adaptation was to include the original practice scenario (“you and your parents are not getting along well”) as an additional test scenario in order to broaden the scope of social relationships focused upon. There were thus 13 scenarios (the practice scenario and 12 test scenarios from the original CSQ) in the first iteration of our revised CSQ (the CSQ-13), which had nine response items for each scenario.

## 3 Results

### 3.1 Results from the 13-item version of the Cognitive Style Questionnaire (CSQ-13)

Possible scores on the CSQ-13 ranged from 117–585. Descriptive statistics for the CSQ-13 are presented in Table 2. Table 3 shows the correlation matrix for relations between scores on the CSQ-13 for the five dimensions of cognitive style (internality, globality, stability, self-worth, and negative consequences). As shown in Table 3, scores for all dimensions were positively correlated with one another. The internal reliability of the scores across the five dimensions was good, *α* = .81. A principal components analysis was performed on the scores for the five dimensions. Kaiser’s (1960) rule, scree-plot analysis, and parallel analysis using a Monte Carlo analysis with 1000 repetitions, all suggested the extraction of a single factor. This factor (with an eigenvalue of 3.08) accounted for 61.65% of the observed variance. All five dimensions loaded onto this factor, with loadings ranging from .35 to .88.

Turning to reliability across the scores for the 13 scenarios, Cronbach’s alpha for the CSQ-13 was .91. As a value of alpha greater than .90 suggests that a questionnaire may contain unnecessary duplication of content (Streiner, 2003), the content of the scenarios on the CSQ-13 was re-examined for item redundancy, leading to the removal of two scenarios (‘low average mark for the year’ and ‘low mark in an assignment’) highly similar to another scenario (‘you receive a low mark for an exam’).

### 3.2 Results from the 11-item version of the Cognitive Style Questionnaire (CSQ-11)

The final 11 scenarios that remained from the CSQ-13 formed the basis of the second version of the CSQ, the CSQ-11, which was administered via the Internet to a separate sample of participants. The response items for the CSQ-11 were identical to those for the corresponding scenarios in the CSQ-13. Possible scores on the CSQ-11 ranged from 99 to 495.

Descriptive statistics for the CSQ-11 are shown in Table 2. Table 4 shows the correlation matrix for relations among scores on the CSQ-11 for the five dimensions of cognitive style (internality, globality, stability, self-worth, and negative consequences). As shown in Table 4, scores for all dimensions were positively correlated with one another. The internal reliability of the scores across the five dimensions was good, *α* = .86. A principle components analysis was performed on the scores for the five dimensions. Kaiser’s (1960) rule, scree-plot analysis, and parallel analysis using a Monte Carlo analysis with 1000 repetitions, all suggested the extraction of a single factor. This factor (with an eigenvalue of 3.31) accounted for 66.15% of the observed variance. All five dimensions loaded onto this factor, with loadings ranging from .52 to .91.

With respect to reliability for scores across the 11 scenarios, Cronbach’s alpha for the CSQ-11 was found to be .89, suggesting that there was still item redundancy (Streiner, 2003). Analyses indicated that the deletion of three scenarios (‘your partner does not want a relationship with you any more’, ‘you do not look as good as you would like in terms of your physical appearance’, and ‘you receive a low mark for an exam’) would reduce the Cronbach’s alpha of the CSQ-11, leaving eight scenarios to form the final version of the CSQ, the CSQ-SF.

### 3.3 Results from the Short-Form version of the Cognitive Style Questionnaire (CSQ-SF)

The final eight CSQ-SF scenarios are presented in Supplementary Material: Appendix 2. As with the CSQ-13 and CSQ-11, each scenario was assessed using nine response items, scored from 1 to 5. Total scores on the CSQ-SF could hence range from 72 to 360, with higher scores reflecting a more negative cognitive style.

Descriptive statistics for the CSQ-SF are reported in Table 2. Table 5 shows the correlation matrix for relations among scores on the CSQ-SF for the five dimensions of cognitive style (internality, globality, stability, self-worth, and negative consequences). As shown in Table 5, scores for all dimensions were positively correlated with one another. The internal reliability of the scores across the five dimensions was good, *α* = .85. A principal components analysis was performed on the scores for the five dimensions. Kaiser’s (1960) rule, scree-plot analysis, and parallel analysis using a Monte Carlo analysis with 1000 repetitions, all suggested the extraction of a single factor. This factor (with an eigenvalue of 3.25) accounted for 65.08% of the observed variance. All five dimensions loaded onto this factor, with loadings ranging from .54 to .89.

### 3.4 Gender differences

On the CSQ-13, women (*M* = 332.36, *SD* = 42.28) scored more highly than did men (*M* = 319.45, *SD* = 43.50), *t*(242) = 2.26, *p* < .025, *d* = 0.30, indicating that women had a more negative cognitive style. There was no difference in CSQ-11 scores between men (*M* = 279.53, *SD* = 32.46) and women (*M* = 283.75, *SD* = 44.02), *t*(388) = 0.98, n.s., *d* = 0.11. There was no difference in CSQ-SF scores between men (*M* = 201.05, *SD* = 28.96) and women (*M* = 197.29, *SD* = 28.65), *t*(276) = 1.09, n.s., *d* = 0.13.

To explore potential reasons for the absence of a gender effect on the CSQ-11 and CSQ-SF, we investigated responses on the original CSQ-13 individual items as a function of gender. Gender differences were observed on only two of the items, with women demonstrating more negative cognitive style in relation to (a) low mark in an assignment, *t*(246) = 3.43, *p* < .001, *d* = 0.46, and (b) not looking good in terms of physical appearance, *t*(246) = 2.54. *p* < .025, *d* = 0.34. The first of these items was omitted in the CSQ-11, and the second was omitted in the CSQ-SF.

### 3.5 Reliability of the CSQ-SF

Reliability across the eight scenarios of the CSQ-SF was good, *α* = 81, being comfortably between the recommended boundaries of 0.7 and 0.9. This showed the CSQ-SF scenarios to have internal reliability. The split-half coefficient was also satisfactory at .78. A principal components analysis was performed on the scores for the eight scenarios. Kaiser’s (1960) rule, scree-plot analysis, and parallel analysis using a Monte Carlo analysis with 1000 repetitions, all suggested the extraction of a single factor. This factor (with an eigenvalue of 3.47) accounted for 43.31% of the observed variance. All eight scenarios loaded onto this factor (with loadings ranging from .46 to .76), suggesting all scenarios similarly assessed cognitive style.

Test–retest reliability over a period of 4 weeks was performed on a sub-sample of 60 of the 276 participants who originally completed the CSQ-SF. The test–retest correlation for total CSQ-SF scores was *r*(58) = .91, *p* < .001. A two-way mixed model intra class correlation with absolute agreement type (Shrout & Fleiss, 1979) found a correlation of .90, *p* < .001. Thus the CSQ-SF demonstrated excellent test–retest reliability.

### 3.6 Validity of the CSQ-SF

Face validity was ensured through the use of a subset of the negative scenarios used in the original CSQ, and response scales addressing the same key dimensions (internal–external, global–specific, stable–unstable, self-worth, negative consequences). Previous studies have shown the negative scenarios of the CSQ to be positively correlated with both the depression and anxiety subscales of the HADS (O’Connor, Connery, & Cheyne, 2000). As shown in Table 6, positive correlations were found between CSQ-SF scores and both the depression and anxiety subscales of the HADS. These relations were maintained when age and gender were controlled for (see Table 6). The fact that more negative cognitive style as assessed using the CSQ-SF was associated with higher scores for depression and anxiety demonstrates the construct validity of the CSQ-SF.

### 3.7 Mode of administration

To investigate possible effects of mode of administration (electronic versus paper-and-pen format), we compared responses to the eight items common to all three versions of the CSQ (those items that formed the CSQ-SF) across the three samples involved. The mean scores for the three versions of the CSQ are shown in Table 7.

Total CSQ scores between samples were compared using one-way ANCOVA with administration mode (electronic, paper-and-pen) as the independent variable and gender and age added as covariates. Comparing scores for the CSQ-13 (paper and pen) with those on the CSQ-11 (electronic), there was no effect of administration mode, *F*(1, 632) = 2.27, n.s., *η*^2^ = .004. Comparing the 8-item scores on the CSQ-11 (electronic) with those on the CSQ-SF (paper-and-pen), there was no main effect of administration mode, *F*(1, 664) = 2.23, n.s., *η*^2^ = .003.

## 4 Discussion

The present article describes the development and validation of a Short-Form version of the Cognitive Style Questionnaire (CSQ-SF). Given that the CSQ-SF may potentially be used as a dependent variable in longitudinal studies, it is often likely to be necessary to retest participants using this measure, raising the possibility that familiarity with the CSQ-SF may act as a confound. However, the excellent test–retest reliability of the CSQ-SF demonstrates its robustness to such a potential confound. The CSQ-SF also showed excellent internal reliability without exhibiting item redundancy, and its split-half reliability was also satisfactory. Factor analysis indicated that the CSQ-SF was unidimensional, with responses for all eight scenarios loading onto one factor. CSQ-SF scores were also related in expected ways with depression and anxiety, with higher scores on the CSQ-SF (indicating more negative cognitive style) correlating positively with those for both depression and anxiety. The CSQ-SF thus appears to have good construct validity.

Our second aim was to establish whether the CSQ could reliably be administered remotely via the Internet. When scores for the electronically-administered CSQ-11 were compared with those for the CSQ-13 and CSQ-SF (both administered in paper-and-pen format), there was no effect of administration mode. These results suggest that the CSQ-SF can reliably be administered in electronic format, and are in line with Jones et al.’s (2008) finding that psychopathology questionnaires are suitable for administration as e-questionnaires.

One issue worthy of further discussion is the fact that, although women were found to have a more negative cognitive style than men on the CSQ-13, this gender difference disappeared for the shorter CSQ-11 and CSQ-SF. The items omitted for the CSQ-11 were ‘low average mark for the year’ and ‘low mark in an assignment’; those additionally omitted for the CSQ-SF were ‘partner no longer wants a relationship with me’, ‘not looking good in terms of physical appearance’, and ‘low exam mark’. Two of these omitted items (low mark in an assignment and not looking good in terms of physical appearance) were the only individual items to show gender differences in the CSQ-13. Thus, the absence of a gender effect on the two shorter versions of the CSQ may be due to the fact that the omitted items are those that are most likely to distinguish between genders. Some support for this suggestion comes from Hankin and Abramson’s (2002) study on adolescents, which found that girls were more likely than boys to rate personal failings as causing negative events. Physical appearance and academic performance are arguably the items most likely to induce explanations that reference personal characteristics, and thus gender differences may be most obvious on these items. To explore this possibility, future research should further investigate how gender relates to cognitive style as a function of the scenario content.

Limitations include the fact that the scenarios in the original CSQ (and thus those employed in the CSQ-SF) were aimed at a student population, and will have less relevance to a more mature adult population. Future research should therefore focus on developing further Short-Form versions of the CSQ appropriate to different age ranges of the general population based on Alloy et al.’s (2001) adaptation. A second limitation is that the CSQ-SF has not yet been shown prospectively to predict depression, as the original CSQ has, and hence its predictive validity has not yet been established. Finally, our study was conducted primarily with undergraduate samples and did not screen participants for psychiatric disorders (reasoned to be unlikely to be prevalent given that these were by definition predominantly high-functioning young adults). It is thus important for future research to establish the reliability and validity of the CSQ-SF when used with patient groups.

In conclusion, we have shown that the CSQ-SF is a reliable and valid measure of negative cognitive style, and is likely to be a useful research tool in this area.

## Figures and Tables

**Table 1:** CSQ Item assessing stability of cognitive style.

Think about the cause (i.e., what you wrote down on the line above) of your receiving a negative evaluation of your job performance. Now assume that in the future, you receive evaluations of your job performance on other occasions. Will the cause of your receiving a negative evaluation of your job performance now as described above again cause you to receive a negative evaluation of your job performance in the future? (Circle one number)								
Will never again cause me to receive negative evaluations of my job performance	1	2	3	4	5	6	7	Will always cause me to receive negative of my job performance

**Table 2:** Descriptive statistics.

Variable	Mean (SD, range)	Cronbach’s alpha
CSQ-13	327.69 (43.03, 226–459)	.91
CSQ-11	282.32 (40.46, 160–416)	.89
CSQ-SF	199.09 (28.81, 113–256)	.81
HADSanxiety	7.91 (4.13, 0–21)	.79
HADSdepression	4.10 (3.51, 0–21)	.79

*Note:* CSQ-13, 13-scenario Cognitive Style Questionnaire; CSQ-11, 11-scenario Cognitive Style Questionnaire; CSQ-SF, 8-scenario Cognitive Style Questionnaire – Short Form; HADSanxiety, Anxiety subscale of the Hospital Anxiety and Depression Scale, HADSdepression, Depression subscale of the Hospital Anxiety and Depression Scale.

**Table 3:** Correlations between the Five dimensions of cognitive style on the CSQ-13.

	Globality	Stability	Self-worth	Negative consequences
Internality	.20⁎⁎	.21⁎⁎	.29⁎⁎	.15⁎
Globality		.73⁎⁎	.67⁎⁎	.66⁎⁎
Stability			.64⁎⁎	.60⁎⁎
Self-worth				.68⁎⁎

⁎ *p* < .025.

⁎⁎ *p* < .001.

**Table 4:** Correlations between the five dimensions of cognitive style on the CSQ-11.

	Globality	Stability	Self-worth	Negative consequences
Internality	.28⁎	.39⁎	.50⁎	.20⁎
Globality		.73⁎	.75⁎	.71⁎
Stability			.74⁎	.63⁎
Self-worth				.64⁎

⁎ *p* < .001.

**Table 5:** Correlations between the five dimensions of cognitive style on the CSQ-SF.

	Globality	Stability	Self-worth	Negative Consequences
Internality	.26⁎	.39⁎	.51⁎	.24⁎
Globality		.71⁎	.69⁎	.71⁎
Stability			.70⁎	.63⁎
Self-worth				.64⁎

⁎ *p* < .001.

**Table 6:** Correlations between CSQ-SF, depression, and anxiety.

	HADSanxiety	HADSdepression
CSQ-SF	.38⁎ (.39⁎)	.28⁎ (.28⁎)
HADSanxiety		.58⁎ (.58⁎)

*Note:* CSQ-SF, Cognitive Style Questionnaire – Short Form; HADSanxiety, Anxiety subscale of the Hospital Anxiety and Depression Scale; HADS depression, Depression subscale of the Hospital Anxiety and Depression Scale. Partial correlations (controlling for age and gender) are shown in parentheses.

⁎ *p* < .001.

**Table 7:** Mean scores on the final eight CSQ items for the three separate samples.

Variable	Mean (SD, range)
CSQ-13 (paper and pen)	198.57 (28.22, 138–284)
CSQ-11 (electronic)	203.18 (30.35, 109–300)
CSQ-SF (paper and pen)	199.09 (28.81, 113–256)

*Note:* CSQ-13, 13-scenario Cognitive Style Questionnaire; CSQ-11, 11-scenario Cognitive Style Questionnaire; CSQ-SF; Cognitive Style Questionnaire – Short Form.
