# Supplementary material for: Assessing negative cognitive style: Development and validation of a Short-Form version of the Cognitive Style Questionnaire
Source: Pers Individ Dif. 2012 Apr;52(5-2):581–5. doi: 10.1016/j.paid.2011.11.026 (PMC3289144; doi:10.1016/j.paid.2011.11.026)
Supplement: Supplementary data 1 [file mmc1.doc]

Appendix 1

Example of Cognitive Style Questionnaire Short Form (CSQ-SF) response format

Imagine you are getting along badly with your parents

Think carefully about the reason for you getting along badly with your parents, then answer the questions below

*Strongly Strongly*

*Agree Agree Undecided Disagree Disagree*

1. Getting along badly with my parents is caused by other people or     

circumstances*

1. The reason I get on badly with my parents causes problems in     

all areas of my life

1. My parents and I will start afresh in the future and forget about the     

reason for getting along badly*

1. Getting along badly with my parents will lead to other negative things     

happening to me

1. Getting along badly with my parents means that there is something     

wrong with me as a person

1. It is my fault if I am getting along badly with my parents     
2. The reason I get on badly with my parents does not stop me from     

enjoying other things*

1. The reason for getting along badly will stop me from getting along     

well with my parents in the future

1. Getting along badly with my parents does not say anything about     

me as a person*

* = item reverse scored

Appendix 2

Scenarios and introduction employed in the Cognitive Style Questionnaire Short Form (CSQ-SF)

Introductory paragraph

“Please try vividly to imagine yourself in each of the situations that follow. Picture each situation as clearly as you can as if the events were happening to you right now. Place yourself in each situation and decide what you feel would have ***caused*** that situation if it actually happened to ***you***. Although the events may have many causes, we want you to choose only one – the thing you feel would be the major cause of the situation if it actually happened to you. It is important to remember that ***there are no right or wrong answers to the questions***. The important thing is to answer the questions in a way that corresponds to what ***you*** would think and feel if the situations occurred in your life.

Scenarios employed

1. Imagine you are getting along badly with your parents.
2. Imagine your class reacts negatively to an important talk you have to give as part of your coursework.
3. Imagine that during the first year of working in the career of your choice, you receive a negative evaluation of your job performance.
4. Imagine you go to a party and people are not interested in you.
5. Imagine you really want to be in an intimate, romantic relationship, but you aren’t.
6. Imagine that in an important class, you can’t complete all the work that your teacher expects of you.
7. Imagine a person you’d really like to develop a close friendship with does not want to be friends with you.
8. Imagine you are unhappy.
